# Practice Effects on Story Memory and List Learning Tests in the Neuropsychological Assessment of Older Adults

**DOI:** 10.1371/journal.pone.0164492

**Published:** 2016-10-06

**Authors:** Brandon E. Gavett, Ashita S. Gurnani, Jessica L. Saurman, Kimberly R. Chapman, Eric G. Steinberg, Brett Martin, Christine E. Chaisson, Jesse Mez, Yorghos Tripodis, Robert A. Stern

**Affiliations:** 1 Department of Psychology, University of Colorado Colorado Springs, Colorado Springs, Colorado, United States of America; 2 Alzheimer's Disease Center, Boston University School of Medicine, Boston, Massachusetts, United States of America; 3 Boston University School of Public Health, Boston, Massachusetts, United States of America; Waseda University, JAPAN

## Abstract

Two of the most commonly used methods to assess memory functioning in studies of cognitive aging and dementia are story memory and list learning tests. We hypothesized that the most commonly used story memory test, Wechsler's Logical Memory, would generate more pronounced practice effects than a well validated but less common list learning test, the Neuropsychological Assessment Battery (NAB) List Learning test. Two hundred eighty-seven older adults, ages 51 to 100 at baseline, completed both tests as part of a larger neuropsychological test battery on an annual basis. Up to five years of recall scores from participants who were diagnosed as cognitively normal (n = 96) or with mild cognitive impairment (MCI; n = 72) or Alzheimer's disease (AD; n = 121) at their most recent visit were analyzed with linear mixed effects regression to examine the interaction between the type of test and the number of times exposed to the test. Other variables, including age at baseline, sex, education, race, time (years) since baseline, and clinical diagnosis were also entered as fixed effects predictor variables. The results indicated that both tests produced significant practice effects in controls and MCI participants; in contrast, participants with AD declined or remained stable. However, for the delayed—but not the immediate—recall condition, Logical Memory generated more pronounced practice effects than NAB List Learning (b = 0.16, p < .01 for controls). These differential practice effects were moderated by clinical diagnosis, such that controls and MCI participants—but not participants with AD—improved more on Logical Memory delayed recall than on delayed NAB List Learning delayed recall over five annual assessments. Because the Logical Memory test is ubiquitous in cognitive aging and neurodegenerative disease research, its tendency to produce marked practice effects—especially on the delayed recall condition—suggests a threat to its validity as a measure of new learning, an essential construct for dementia diagnosis.

## Introduction

Alzheimer’s disease (AD) is a neurodegenerative disease characterized by early and progressive decline in episodic memory due to medial temporal lobe pathology [1,2]. Episodic memory refers to the ability to learn and recall personal experiences, whereas semantic memory is a more stable representation of factual knowledge [3]. Recent research suggests that AD pathology begins to accumulate decades before clinical symptoms become apparent [4]. When the earliest clinical symptoms of AD appear, they typically involve isolated episodic memory deficits that do not affect functional independence; in this case, a diagnosis of mild cognitive impairment (MCI) is appropriate. Even as the clinical presentation of AD progresses from MCI to dementia, recall of highly rehearsed material and memories from long ago are more likely to remain intact compared to newly learned information and recent events, which are likely to be rapidly forgotten [5]. As such, cognitive tests that evaluate an individual's ability to learn new information, as opposed to one's ability to retrieve more stable remote memories, are more sensitive to AD, especially in its early stages [6]. This speaks to the importance of using cognitive assessment measures that are novel to the examinee when measuring new learning.

Two of the most popular methods for measuring new learning in the verbal/auditory modality are the list learning and story memory paradigms [7]. These methods are commonly used in the assessment of older adults—especially those with MCI and AD—to identify episodic memory impairment and track changes in new learning over time [8–12]. Within the past half century, numerous verbal list learning tests have been developed, such as the California Verbal Learning Test (CVLT) [13], Hopkins Verbal Learning Test (HVLT) [8], Word List Recall test from the Consortium to Establish a Registry for Alzheimer’s Disease (CERAD) [14], and Rey Auditory Verbal Learning Test (RAVLT) [15]. Most list learning tests provide recall scores for each learning trial, as well as a sum of recall across immediate learning trials; one or more delayed recall scores at various intervals (e.g., short and long delay); and a yes/no recognition score. The list learning paradigm, in its various formats, has been shown to distinguish between healthy aging, MCI, and AD with good sensitivity and specificity [6,16–20]. Consistent with these findings, AD pathology has been shown to be associated with poorer performance on list learning tests in comparison to pathology free individuals [21].

One list learning test, the Neuropsychological Assessment Battery (NAB) List Learning test [22], uses three learning trials of 12 words that can be organized into three semantic categories. This allows for the assessment of semantic clustering along with immediate free recall, short- and long-delayed free recall, recognition, intrusions, and repetitions. It has been shown to have classification accuracies similar to or better than other list learning measures and utility for predicting the clinical course and diagnostic outcomes of cognitively normal, MCI, and AD individuals [10,11].

In comparison to the variability among the multitude of list learning tests available to neuropsychologists, there are fewer tests of story memory available, with one test that predominates: the Logical Memory (LM) subtest from the various Wechsler Memory Scale (WMS) editions [23–26]. On this test, examinees are read one or two stories and are asked to recall them immediately and again after a delay. Wechsler's Logical Memory subtest has been shown to effectively distinguish between AD, MCI, and healthy controls [27,28] and has been consistently used as a measure of episodic memory in large-scale longitudinal studies [12,29–31]. A number of studies have compared story memory with list learning and found that list learning tests possess better sensitivity for distinguishing between healthy controls, MCI, and AD, and for predicting the rate of conversion from MCI to AD [6,32–34]. In addition, list learning tests that require active organization strategies to cluster the stimuli into meaningful categories (e.g., CVLT-II, NAB List Learning) are more susceptible to executive functioning deficits than stimuli that are presented in a logically organized fashion, such as Logical Memory [35]. As such, a logically organized story may be easier to encode than an unorganized list of words, especially for individuals with executive functioning difficulties.

Serial assessment is often used to monitor disease progression in the course of neurodegenerative conditions such as AD. Data from multiple evaluation points can assist with differential diagnosis, as criteria for AD and other dementias require a decline in cognition from a previous ability level [36]. In theory, normative data can be used to interpret an examinee’s performance in relation to age-matched peers; however, normative data are often collected at only one time point, making it difficult to justify their use in the context of serial assessments [37–41]. In order to accurately interpret the meaning of a change in score between two or more time points, clinicians must be aware of critical factors that can impact scores on subsequent assessments such as regression to the mean, the reliability of the measure, practice effects, and maturation effects (e.g., aging) [39]. Because of the complexity of interpreting change on serial assessments, several guidelines and methods have been proposed, such as various reliable change models and standardized regression-based methods [42–44]. Despite the importance of this issue, there is still a dearth of research examining performance trends across serial assessments.

Practice effects are conceptualized as the amount of improvement expected to occur with repeated exposure to a test [43]. Age, education level, disease status, and the characteristics of the test itself can all influence the magnitude of practice effects [39,45]. Practice effects can occur differentially over multiple testing points and have been described as having the most impact on the first two retests [46]. Some research has found that practice effects within the memory domain (as measured by both a list learning and story recall task) may be present between baseline and second testing, followed by a sharp decline on subsequent evaluations in normal individuals who eventually convert to mild cognitive impairment or AD [47]. However, other research has suggested that the absence of practice effects may be a good indicator of preclinical dementia [48,49]. Because of the emphasis on longitudinal assessment in dementia research (e.g., the Alzheimer's Disease Centers' Uniform Data Set, the Alzheimer's Disease Neuroimaging Initiative), it is essential to properly characterize the expected practice effects on various instruments that are commonly used in dementia assessment and to understand how clinical diagnosis (e.g., control, MCI, AD) affects scores obtained via serial assessment.

Although the Logical Memory subtest has been shown to be a valid marker of medial temporal lobe dysfunction [30,50] and has adequate inter-rater reliability [51], its test-retest reliability and associated practice effects are an important limitation to its longitudinal application [39]. The Logical Memory subtest has been shown to produce considerable practice effects, even with the use of alternate forms [52]. For instance, Gavett et al. [39] examined reliable change on several neuropsychological tests over multiple visits and found large practice effects (0.84 points per year for immediate recall and 1.10 points per year for delayed recall) for the Logical Memory subtest that substantially outweighed associated maturation effects.

One method used to attenuate practice effects is the use of alternate forms. Alternate forms are variations on a test that have been determined to be relatively equal through the process of test development and norming [53]. Historically, it has been more common for alternate forms of a test to be developed for list learning tests than story memory tests. For example, alternate forms are available for the NAB List Learning Test, CVLT-II, RAVLT, and HVLT-R, among others. In contrast, alternate forms are not typically available for the Wechsler Logical Memory test. Practice effects have been observed using the alternate form of the CVLT-II (Cohen's d range = -0.01 to 0.18), but were less pronounced than when the standard form was used at the first and second testing (Cohen's d range = 0.27 to 0.61) [54]. Significant practice effects on the CVLT were detected but controlled for using a dual baseline procedure in a sample of HIV-positive participants [55]. Although there is evidence for practice effects on list learning tasks, one study [56] found that a list learning task was one of the least susceptible to these effects compared to other tasks in a comprehensive battery in those with and without brain injury across 20 closely spaced assessments.

Because of the regularity with which older adult research participants undergo memory assessment, repeated exposure to the same Logical Memory narrative may cause considerable practice effects that reduce the validity of the test as a measure of new learning. Consequently, reductions to the validity of the Logical Memory story may have unintended consequences for application of inclusion and exclusion criteria for AD research projects. The goal of the current study is to compare the practice effects produced on the two episodic memory paradigms reviewed above—list learning and story memory—in a sample of older adults diagnosed as cognitively normal, MCI, or AD. The list learning test used in the current study is from the NAB, whereas the story memory test used in the current study is the WMS-R version of Logical Memory (Story A only). Due to the fact that the content of the Logical Memory test is organized logically, and because it does not employ alternate forms, we hypothesized that this test will produce larger practice effects than the NAB List Learning Test, which is unorganized and uses alternate forms. Further, we hypothesized a dose-response relationship between diagnosis and practice effects, such that—on both memory tests—controls will exhibit the most pronounced practice effects, followed by those with MCI; participants with AD are expected to exhibit the least pronounced practice effects.

## Materials and Methods

### Participants and Procedure

The Boston University Medical Campus Institutional Review Board approved this study. Participants were volunteers in the longitudinal research registry of the Boston University (BU) Alzheimer's Disease Center (ADC), which is one of the 34 past and present ADCs nationwide funded by the National Institute on Aging. The registry uses clinician referrals, community outreach (e.g., lectures, presentations), and word-of-mouth to recruit cognitively healthy individuals as well as those with MCI and AD dementia. All participants provided written consent to have their data used for research purposes, in accordance with the Declaration of Helsinki. Participants completed annual study visits, which include a comprehensive neuropsychological assessment battery along with a detailed neurological examination and gathering of social, medical, and family history. A more detailed description of the registry has been published previously [57].

For the current study, we began with archival data collected from January 03, 2005 to February 15, 2016. The initial data set was made up of 689 participants who participated in a total of 3703 study visits. At each study visit, participants were assigned a clinical diagnosis by a consensus team of experts made up of neurologists, neuropsychologists, psychiatrists, nurse practitioners, and research assistants. All clinical diagnoses were made according to commonly accepted criteria, with one exception. The Petersen criteria [58,59] were used to diagnose MCI; however, individuals with no self or informant complaint, but with objective cognitive impairment, were also considered to have MCI for the current study. The National Institute of Neurological and Communicative Disorders and Stroke—Alzheimer's Disease and Related Disorders Association (NINCDS-ADRDA) criteria were used to diagnose participants with AD [60].

**Fig 1:**
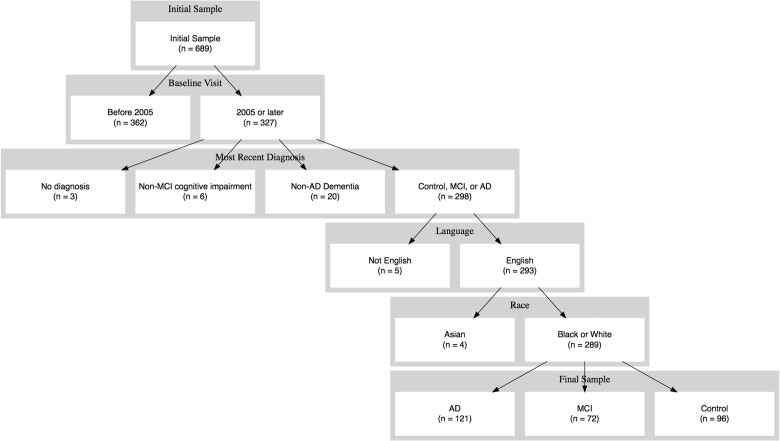
Participant Flow. Flowchart illustrating application of inclusion and exclusion criteria for the current study.

Because the BU ADC had been enrolling participants prior to the establishment of the current data collection procedures, some participants had previous exposure to neuropsychological testing. Therefore, we excluded participants whose baseline visit occurred prior to initiation of the current protocol, so that each participant's baseline visit corresponded to their first known exposure to the two memory tests used in this study. We sought to include participants who—at their most recent study visit—were given a consensus diagnosis of control, MCI, or AD (either Possible or Probable). Therefore, we excluded participants who, at their last study visit, were either diagnosed with a non-AD etiology for dementia or who exhibited subtle cognitive difficulties that were not sufficient to meet criteria for MCI. We also excluded individuals whose primary language was not English in order to eliminate any potential language confounds. The vast majority of participants described themselves as either Black/African American or White/Caucasian. Therefore, we excluded four Asian participants so that analyses about race would be restricted to the two majority groups in this sample. The flowchart in Fig 1 depicts the application of these inclusion and exclusion criteria to generate the sample that was used for all subsequent analyses. Finally, because few participants completed more than five annual study visits, we restricted our analyses to the first five visits.

### Neuropsychological Measures

For the current study, two different measures of verbal episodic memory were administered to participants at each study visit. These two measures were used to test the hypothesis that story memory tests are more susceptible to practice effects than list learning tests. The story memory test used was Logical Memory Story A from the Wechsler Memory Scales—Revised (WMS-R) [24]. The list learning instrument was the NAB List Learning Test [22].

#### WMS-R Logical Memory Story A

In the Logical Memory test, participants are read a logically organized story and asked to recall the story immediately after its presentation (Immediate Recall). Approximately 20 minutes later, the participants are again asked to recall the story from memory (Delayed Recall). The version used in this study uses only one story (Story A) read once to participants at each study visit. This procedure is based on those used across all ADCs following the National Alzheimer’s Coordinating Center’s Uniform Data Set [31]. Possible scores for both Logical Memory Immediate and Delayed Recall trials range from 0 to 25, with higher scores reflecting more details recalled.

#### NAB List Learning

In the NAB List Learning Test, participants are read a list of 12 words that can be organized into three different semantic categories and asked to recall as many of those words as possible. The list of words is repeated a second and third time, with recall trials immediately following each presentation. The Immediate Recall total score is the sum of all words recalled across the three learning trials. After the three learning trials, a second list of words is presented that has partial overlap with the categories embedded within the first list of words. After recall of this distractor list, participants are asked to spontaneously recall the words from the first list (Short Delay Free Recall). Later, after a delay of approximately 12 minutes, participants are again prompted to spontaneously recall the first list of words (Long Delay Free Recall). Finally, a yes/no paradigm is used to evaluate recognition memory. For the current study, we use the Immediate Recall (range = 0–36) and Long Delay Free Recall (range = 0–12) scores as the primary scores from the NAB List Learning Test, as these are most compatible with the Immediate and Delayed Recall scores generated by Logical Memory. For both NAB List Learning scores, higher values reflect more words recalled. Importantly, the NAB List Learning Test has an alternate form; in the current study, participants alternated between the two forms at each study visit as method for attenuating practice effects.

### Data Analysis

To test the hypothesis that story memory causes more pronounced practice effects than list learning, we used linear mixed effects regression. Linear mixed effects models provide a flexible framework for evaluating model fit, estimating population parameters, and accounting for the hierarchical nature of longitudinal data. We performed two separate linear mixed effects models, one for the immediate recall condition (Logical Memory Immediate Recall and NAB List Learning Immediate Recall) and one for the delayed recall condition (Logical Memory Delayed Recall and NAB List Learning Long Delay Free Recall). In each model, fixed effects predictor variables included age at baseline (centered), sex (male vs. female), years of education (centered), race (White/Caucasian vs. Black/African American), time since baseline visit (years), visit number (1–5), test (Logical Memory vs. NAB List Learning), and diagnostic group (control, MCI, or AD). We also modeled interaction effects between visit number, test, and diagnostic group in order to determine if the latter two variables are associated with differential rates of change (practice effects) over time.

Because the Logical Memory and NAB List Learning Test raw scores are scaled differently, we converted them to z-scores to promote direct comparisons. We derived the z-scores for each test using the means and standard deviations of the control participants at baseline as the standard for comparison.

In addition to the fixed effects described above, we added random intercept and slope terms to the regression models, which allow for inter-individual variability in baseline performance (intercept) and rate of change over time (slope). We analyzed the data using linear mixed effects modeling with restricted maximum likelihood estimation in R software version 3.2.4 [61] and its lme4 package version 1.1–10 [62]. To perform hypothesis tests on the fixed effects parameter estimates, we used the Satterthwaite approximation [63] to estimate the appropriate degrees of freedom, as implemented in R's lmerTest package version 2.0–29 [64].

## Results

**Fig 2:**
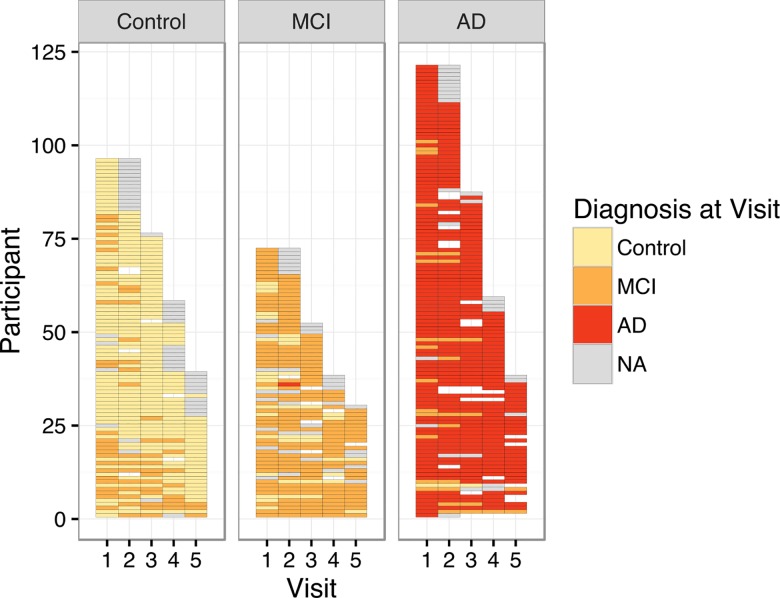
Clinical Diagnosis by Visit. Heatmap depicting the clinical diagnosis assigned to each participant at each study visit. The left panel represents participants whose most recent diagnosis was Control. The middle panel represents participants whose most recent diagnosis was MCI. The right panel represents participants whose most recent diagnosis was AD. Colors reflect the diagnosis made at a given visit, which does not always correspond to the most recent diagnosis. Participants' most recent visit may have occurred beyond the 5 visits used in the current study.

**Fig 3:**
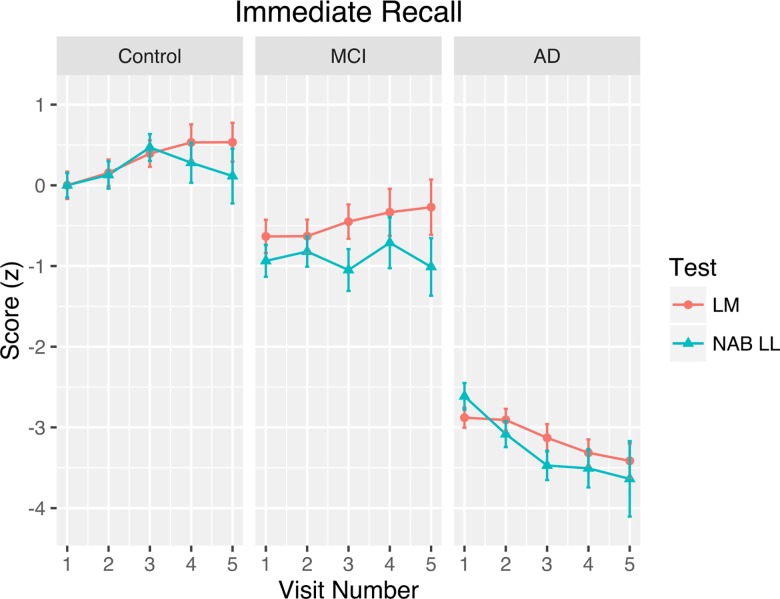
Immediate Recall Practice Effects. Standardized test scores on the immediate recall condition as a function of visit number, test, and clinical diagnosis. Error bars represent 95% confidence intervals and account for within-subjects variability.

Participant age at baseline ranged from 51 to 100. Years of education ranged from 3 to 21. The median number of study visits completed was 4. A more detailed breakdown of the baseline demographic characteristics of the current sample is presented in Table 1. A graph depicting the clinical diagnosis that was assigned to participants at each study visit is shown in Fig 2. To ensure that there were no between-groups differences in interval from baseline to any of the five follow-ups, linear mixed effects regression was used to examine the effect of the interaction between group and visit number on the duration of the assessment interval. There were no significant differences in the test-retest interval for any of the visits when comparing the control group to the MCI (b = 0.009, SE = 0.029) and AD (b = 0.037, SE = 0.026) groups. The immediate recall data, shown as a function of test, visit number, and diagnosis, are plotted in Fig 3. The model for immediate recall yielded random effects standard deviations of 0.71 for the intercept, 0.03 for the slope, and 0.74 for the residual. The fixed effects parameter estimates for the immediate recall data are presented in Table 2.

**Table 1:** Baseline Demographic Characteristics of the Current Sample.

Variable	Total Sample	Control	MCI	AD
n	289	96	72	121
Age, years; M (SD)	74.36 (8.08)	71.67 (7.6)	73.29 (7.02)	77.12 (8.22)
Education, years; M (SD)	15.3 (2.96)	16.03 (2.69)	15.58 (2.79)	14.55 (3.11)
Visits, n; M (SD)	4.48 (2.53)	4.77 (2.68)	5.04 (3.09)	3.93 (1.87)
Visits; range	2–12	2–12	2–11	2–9
MMSE; M (SD)	25.07 (7.08)	29.02 (1.11)	28.28 (1.49)	20.03 (8.59)
LM-I; M (SD)	9.83 (5.7)	14.09 (3.37)	11.96 (3.74)	4.39 (3.72)
LM-D; M (SD)	8.36 (6.17)	13.2 (6.17)	11 (3.8)	2.19 (3.32)
LL-I; M (SD)	17.73 (6.68)	22.59 (4.18)	18.68 (4.79)	11.68 (5.06)
LL-D; M (SD)	4.77 (3.74)	7.92 (2.37)	5.04 (2.64)	1.09 (1.83)
Sex, Female; n (%)	144 (49.83%)	56 (58.33%)	43 (59.72%)	45 (37.19%)
Race, Caucasian; n (%)	235 (81.31%)	83 (86.46%)	44 (61.11%)	108 (89.26%)
CDR; n (%)			
0	136 (47.72%)	84 (87.5%)	50 (71.43%)	2 (1.68%)
0.5	73 (25.61%)	12 (12.5%)	20 (28.57%)	41 (34.45%)
1	52 (18.25%)	0 (0%)	0 (0%)	52 (43.7%)
2	8 (2.81%)	0 (0%)	0 (0%)	8 (6.72%)
3	16 (5.61%)	0 (0%)	0 (0%)	16 (13.45%)

Diagnostic groups (Control, MCI, AD) are based on consensus diagnosis at participants' most recent visit, whereas the data in this table are from participants' baseline visit. MCI = mild cognitive impairment; AD = Alzheimer's disease; MMSE = Mini-Mental State Examination; LM = Logical Memory; I = Immediate; D = Delayed; LL = List Learning; CDR = Clinical Dementia Rating.

**Table 2:** Results of the Linear Mixed Effects Model for Immediate Recall.

Parameter	b	SE	df	t	p
Intercept	-0.74	0.18	1118.69	-4.08	< 0.01
Age at baseline	-0.02	0.01	274.63	-2.28	0.02
Sex	0.36	0.11	265.05	3.38	< 0.01
Education	0.09	0.02	269.36	4.75	< 0.01
Race	0.08	0.14	265.84	0.53	0.60
Time (years) since baseline	-0.17	0.11	1000.16	-1.56	0.12
Visit	0.39	0.12	982.57	3.19	< 0.01
Test	0.13	0.13	1302.9	1.02	0.31
DX1	-0.46	0.19	584.14	-2.43	0.02
DX2	-2.08	0.18	591.63	-11.84	< 0.01
Visit x Test	-0.08	0.05	1302.35	-1.72	0.09
Visit x DX1	-0.14	0.06	1080.46	-2.46	0.01
Visit x DX2	-0.45	0.05	1110.03	-8.51	< 0.01
Test x DX1	-0.27	0.21	1302.79	-1.3	0.20
Test x DX2	0.06	0.19	1310.64	0.32	0.75
Visit x Test x DX1	-0.02	0.07	1302.57	-0.34	0.74
Visit x Test x DX2	-0.04	0.07	1307.1	-0.64	0.52

SE = standard error; DX1 = MCI vs. Control; DX2 = AD vs. Control; Logical Memory is the reference group for the Test variable.

As can be seen in Table 2, there were a number of main effects and interactions influencing immediate recall performance. Younger age, female sex, and higher education were associated with better immediate recall scores. The effect of time since baseline visit was not a significant predictor of immediate recall performance. As expected, participants diagnosed with MCI and AD recalled substantially less than controls. In addition, noticeable practice effects were found, such that each visit was associated with a 0.39 standard deviation increase in the immediate recall performance of controls. Although the learning slope in MCI participants was significantly lower than in controls, it was nevertheless sizeable in effect (0.26 standard deviations per year). The AD group, on the other hand, showed a decline (-0.06 standard deviations per year) in performance over time. Finally, there was no main effect of test (Logical Memory vs. NAB List Learning) on overall immediate recall, nor did test interact with visit number or diagnosis to suggest any differential practice effects for Logical Memory vs. NAB List Learning on immediate recall (see Fig 3).

**Fig 4:**
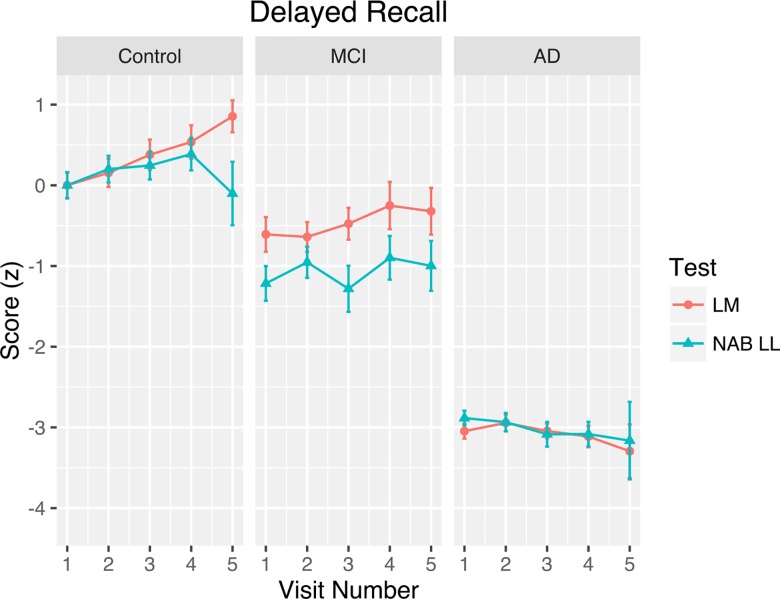
Delayed Recall Practice Effects. Standardized test scores on the delayed recall condition as a function of visit number, test, and clinical diagnosis. Error bars represent 95% confidence intervals and account for within-subjects variability.

The delayed recall data, shown as a function of test, visit number, and diagnosis, are plotted in Fig 4. The model for delayed recall yielded random effects standard deviations of 0.7 for the intercept, 0.08 for the slope, and 0.69 for the residual. The fixed effects parameter estimates for the delayed recall data are presented in Table 3.

**Table 3:** Results of the Linear Mixed Effects Model for Delayed Recall.

Parameter	b	SE	df	t	p
Intercept	-0.83	0.17	768.89	-4.97	< 0.01
Age at baseline	-0.02	0.01	286.6	-3.07	< 0.01
Sex	0.33	0.09	275.7	3.58	< 0.01
Education	0.08	0.02	280.22	4.87	< 0.01
Race	0	0.12	266.82	0.03	0.98
Time (years) since baseline	-0.21	0.1	567.26	-2.06	0.04
Visit	0.47	0.11	512.26	4.13	< 0.01
Test	0.28	0.12	1167.65	2.28	0.02
DX1	-0.35	0.18	510.48	-1.94	0.05
DX2	-2.36	0.17	524.78	-14.24	< 0.01
Visit x Test	-0.16	0.04	1167.13	-3.8	< 0.01
Visit x DX1	-0.17	0.05	363.91	-3.32	< 0.01
Visit x DX2	-0.32	0.05	424.56	-6.4	< 0.01
Test x DX1	-0.74	0.19	1169.2	-3.83	< 0.01
Test x DX2	-0.2	0.18	1190.18	-1.1	0.27
Visit x Test x DX1	0.12	0.07	1170.01	1.74	0.08
Visit x Test x DX2	0.14	0.07	1189.55	2.19	0.03

SE = standard error; DX1 = MCI vs. Control; DX2 = AD vs. Control; Logical Memory is the reference group for the Test variable.

The data in Table 3 reveal a number of main effects and interactions influencing delayed recall performance. Similar to the results for the immediate recall condition, younger age, female sex, and higher education were all associated with better delayed recall scores. In contrast, maturation effects (time since baseline), which were not a significant predictor of immediate recall scores, did have an influence on delayed recall scores, producing a decline of approximately -0.21 standard deviations per year. As above, noticeable practice effects were found; however, the delayed recall results differed from the immediate recall results in that the test variable (Logical Memory vs. NAB) was associated with different patterns of performance across visits. The three-way interaction between visit, test, and diagnosis reveals that controls and AD participants differ significantly when comparing the practice effects produced by each test, whereas MCI participants did not differ from controls. When controlling for all other covariates, the data reveal learning slopes in controls of 0.47 for Logical Memory and 0.31 for the NAB List Learning Test; learning slopes in MCI participants of 0.3 for Logical Memory and 0.25 for the NAB List Learning Test; and learning slopes in AD participants of 0.15 for Logical Memory and 0.13 for the NAB List Learning Test (Fig 3).

As can be seen in Table 1, most of the participants in the AD group were already suffering from cognitive impairment at their baseline visit. Therefore, it may be possible that floor effects on the two memory tests interfered with our ability to observe either practice effects or more pronounced cognitive decline in this group. To further explore this possibility, we excluded our analyses to the AD group only and divided this sample into two subsamples based on a median split of MMSE scores at baseline. We then examined trends in Immediate and Delayed recall on Logical Memory and NAB List Learning as a function of baseline MMSE, using mixed effects regression models similar to those described above. The median baseline MMSE value was 23; the low and high baseline MMSE groups were therefore composed of AD participants with baseline MMSE scores of ≤ 23 (n = 66) and > 23 (n = 55), respectively. On the Delayed Recall trial, there was a significant interaction between visit number and baseline MMSE group, b = -0.107, SE = 0.051, such that AD participants with MMSE scores of > 23 at baseline showed more rapid decline on Delayed Recall than AD participants with MMSE scores of ≤ 23 at baseline. Although the interaction term for Immediate Recall was nearly identical, (-0.109), this estimate was less precise (SE = 0.073) and therefore did not achieve statistical significance.

## Discussion

Story recall and list learning are two of the most common methods used in clinical research to measure verbal episodic memory [7]. In particular, the Wechsler Logical Memory test is frequently used to determine eligibility for research studies related to AD and other dementias. Users of the test should keep in mind that the story's main character would be 71 years old [65] at the time this study was conducted, and because her story has changed relatively little since its inception, it is likely that many patients and research participants have had some exposure to her plight. The ubiquity with which this test is administered to older adults, and the role that this test has in determining diagnosis, study eligibility, and monitoring treatment outcomes [29], all highlight the need to understand the practice effects that occur on this test. A previous study examining longitudinal outcomes in a national sample of cognitively healthy older adults showed that Logical Memory Immediate and Delayed recall produced substantial practice effects with repeated exposure to the story [39]. Therefore, the goals of the current study were twofold: 1) to extend the previous findings to individuals with MCI and AD and 2) to directly compare practice effects on Logical Memory to practice effects on the NAB List Learning Test. We hypothesized that, due to its logically organized structure [35] and lack of alternate forms, Logical Memory would produce more pronounced practice effects than the NAB List Learning Test across all groups (control, MCI, AD).

The results of this study only partially supported our hypotheses. Although practice effects were pronounced—in controls and MCI patients—on both immediate and delayed recall conditions, the format of the test (story vs. list) only served to moderate practice effects in the delayed recall condition. For immediate recall, linear rates of change across visits did not differ based on the test administered. In the delayed recall condition, however, the hypothesized difference in practice effects based on test was observed. In controls, there was a large practice effects discrepancy between tests; the practice effects discrepancy in the MCI group was smaller but not significantly different from controls. In contrast, the practice effects discrepancy in the AD group was found to significantly differ from controls, suggesting that diagnosis moderates the effect of test on learning slope with repeated exposure to a stimulus. In particular, individuals with better memory are more susceptible to exhibiting practice effects over time on delayed recall of Logical Memory compared to NAB List Learning, whereas individuals with memory impairment due to AD do not perform differently over time on the two delayed recall tasks.

One possible factor that may have influenced these findings is the potential for floor effects to have obscured relevant trends in the recall performance of individuals in the AD group, most of whom entered the study having already experienced cognitive decline. Analysis of recall trends in the AD group only, using baseline MMSE grouping as a predictor variable, was performed to examine the potential influence of floor effects on the most impaired subset of our sample. The results indicated that these floor effects may have prevented us from observing even more rapid decline in the AD sample than was possible given the difficulty of the two memory tests used here. Had our sample of AD participants been less impaired at baseline, the primary analyses may have shown more decline, rather than seeming stability, in recall scores across visits. A less impaired sample at baseline would have likely magnified the observed differences in practice effects between the AD group and the other two groups.

Because our model included age at baseline, number of visits, and time (years) since baseline as predictors, the current results demonstrate that the practice effects that are elicited by repeated exposure to tests of verbal episodic memory are—for controls and patients with MCI—more powerful than the decline in episodic memory that occurs as part of the aging process. These findings are consistent with previous work showing that tests of both episodic and semantic memory are affected more strongly by repeated exposure to a stimulus (i.e., practice effects), whereas tests of attention and executive functioning are affected more strongly by maturation (i.e., aging) [39]. This pattern was not observed in participants with AD, however, which indicates that the underlying disease process exerts a more powerful influence over test results than repeated exposure to a test. Such a conclusion is consistent with previous data suggesting that the absence of practice effects may be an important clinical marker for underlying pathology [48,49].

These results conflict with other research that has suggested that practice effects level off after two to three exposures to a test [46]. Although we only modeled our data using a linear trend, a visual inspection of the data presented in Figs 3 and 4 reveals a continued effect of practice beyond the second and third visits. Some experts have recommended employing a prebaseline assessment period whereby participants are exposed to the tests prior to the baseline visit in order to minimize practice effects (e.g., [55,66]). Although our results did not seek to examine the effectiveness of this recommendation, per se, our findings of continued practice effects beyond the first two to three visits may suggest that—at least for episodic memory tests—it is unlikely that practice effects can be eliminated entirely.

Because control participants—and, to a lesser extent, those with MCI—exhibited such pronounced practice effects, there is a convincing argument to be made that all episodic memory measures should be normed with not just cross-sectional data, but with longitudinal data as well. Given the importance of serial neuropsychological assessment in the elderly for the purposes of differential diagnosis, treatment monitoring, clinical trial efficacy, and so forth, it is not advised to rely on cross-sectional normative data to interpret results obtained via serial assessment. At the very least, data focusing on test-retest reliability and practice effects are essential for interpreting reliable change [42,44].

Due to the retrospective nature of this study, one important confound remains unaccounted for. As discussed above, the NAB List Learning Test used alternate forms, whereas the Logical Memory test did not. Therefore, the results of this study cannot disentangle the paradigmatic differences between story memory and list learning. It is possible that these results simply reflect the relative differences between using and not using alternate forms of a test. On the other hand, the inherent organization of the Logical Memory task may make it more amenable to practice effects. Further research is needed to differentiate the role of alternate forms in attenuating practice effects using the same learning paradigm. We can only conclude from the current study that the Wechsler Logical Memory test, which lacks alternate forms, produced more pronounced practice effects on delayed recall in controls and MCI participants than when compared to the alternate forms of the NAB List Learning Test. It is also important to note that the retrospective nature of the current study only allowed for the analysis of two different memory tests. As such, these results may not apply to other test of episodic memory.

Other limitations are as follows: The sample was primarily recruited through convenience methods in a geographically restricted area. Similarly, the sample was highly educated and lacking in racial diversity. Therefore, the external validity of the study may be limited. Because only controls and participants with MCI and AD were included, the results cannot be generalized outside of those diagnostic groups. Similarly, the MCI group in the current sample included participants who demonstrated objective cognitive impairment, but not all of these participants presented with a self- or informant complaint of cognitive difficulties. Therefore, the current results may not generalize to participants diagnosed with MCI using the requirement of a cognitive complaint [59]. Further, the diagnostic groups were defined based on clinical diagnosis without pathological confirmation. Another limitation of the study is that not all participants completed five study visits, either due to attrition or because participants enrolled less than four years ago. Therefore, the follow-up data have been obtained from a highly selected sample and may also lack external validity. In addition, the results may be biased by the fact that the Logical Memory and NAB List Learning Tests were used for clinical diagnosis; this potential bias is likely to affect the analyses related to the three different diagnostic groups (control, MCI, AD), but is not likely to produce confounds related to the study's primary results, which show that the Logical Memory is associated with more pronounced practice effects on delayed recall than NAB List Learning. Finally, the current results are exclusively based on group data, and may not be applicable for individual-level decision-making. Future research should seek to determine the diagnostic accuracy of practice effects data across multiple visits for clinical decision-making at the individual level.
